# Biosynthetic Nano-Selenium by *Bacillus licheniformis* Enhances Growth and Health of Largemouth Bass (*Micropterus salmoides*)

**DOI:** 10.4014/jmb.2508.08049

**Published:** 2025-12-19

**Authors:** Xiaohu Li, Ge Bai, Lifei Feng, Honghao Ding, Keke Li, Wen Zhou, Yunxiang Liang, Yingjun Li

**Affiliations:** 1 National Key Laboratory of Agricultural Microbiology, College of Life Science and Technology, Huazhong Agricultural University, Wuhan 430070, P.R. China; 2 Henan Jinbaihe Biotechnology Co., Ltd., Tangyin, Anyang 455000, P.R. China; 3 State Key Laboratory of Green Pesticide, Central China Normal University, Wuhan 430079, P.R. China

**Keywords:** Selenium sources, nano-selenium, *Bacillus licheniformis*, largemouth bass, antioxidant capacity, intestinal microbiota

## Abstract

Selenium is an essential micronutrient in aquaculture, with its efficacy and safety strongly dependent on the chemical form used for supplementation. In this study, we systematically compared the effects of three selenium sources—sodium selenite (SS), selenium-enriched *Bacillus licheniformis* fermentation broth (BlSe), and selenium-enriched *Saccharomyces cerevisiae* fermentation broth (ScSe)—on the growth performance and physiological parameters of largemouth bass (*Micropterus salmoides*). The SS group exhibited a survival rate of 90%, whereas both the BlSe and ScSe groups achieved 100% survival. Compared with the control, the ScSe group showed a significant increase in weight gain rate. In contrast, the BlSe group displayed markedly reduced serum levels of alanine aminotransferase (ALT), aspartate aminotransferase (AST), and alkaline phospholipase (ALP). All selenium-supplemented groups demonstrated substantially enhanced muscle selenium content—by 326% (SS), 455% (BlSe), and 88% (ScSe)—together with elevated activities of hepatic glutathione peroxidase (GSH-Px) and total superoxide dismutase (T-SOD), and reduced malondialdehyde (MDA) content. Intestinal microbiota analysis revealed a shift from Firmicutes dominance (50%) in the control to Proteobacteria dominance (60%) across all treatment groups at the phylum level. At the genus level, *Mycoplasma* was predominant in the control and BlSe groups (~20% abundance), while *Sphingomonas* and *Chloroplast* were enriched in the SS and ScSe groups, respectively. Moreover, KEGG pathway analysis indicated upregulation of taurine and hypotaurine metabolism, biosynthesis of unsaturated fatty acids, and fatty acid biosynthesis in all selenium-treated groups. These findings underscore the potential of *B. licheniformis*-derived nano-selenium as a highly effective selenium source for sustainable aquaculture.

## Introduction

As a key component of glutathione peroxidase and other selenoproteins, selenium is an essential trace element for maintaining animal health. However, its dietary concentration must be carefully balanced, as both deficiency and excess can negatively impact growth and reproductive performance [1, 2]. Although selenium toxicity is relatively uncommon in aquaculture, deficiency is frequently observed and poses a significant constraint on production [3]. Therefore, appropriate supplementation is necessary when natural dietary and environmental selenium is insufficient [4]. Inorganic forms of selenium, such as selenate and selenite, are highly soluble but exhibit considerable toxicity even at low concentrations [5]. In contrast, organic selenium (*e.g.*, selenomethionine in selenium-enriched yeast) and bio-synthesized nano-selenium are generally considered safer and more effective for dietary supplementation in aquaculture [6, 7]. The representative organic selenium compounds include selenium yeast (Se-yeast), selenomethionine (SeMet), hydroxyselenomethionine (OH-SeMet), and methylselenomethionine (SeMCys). Organic selenium can be efficiently absorbed by the animal body through amino acid transport channels and participate in protein synthesis. When the human body needs selenium, this protein can slowly release selenium. Nano-selenium refers to elemental selenium at the nanoscale. Compared with elemental selenium, nano-selenium has a smaller particle size, making it easier to be absorbed and utilized. It is mainly prepared through biotechnological methods [8].

Selenium yeast (Se-yeast), a widely used organic selenium source, has been shown to improve growth performance, feed efficiency, and glutathione peroxidase (GSH-Px) activity in species such as Nile tilapia (*Oreochromis niloticus*)[9]. Notably, studies indicate that a lower dietary inclusion of Se-yeast (0.2 mg Se/kg) can achieve growth and survival rates comparable to those obtained with higher doses of sodium selenite (0.4 mg Se/kg) [10]. Dietary supplementation with selenium-enriched *Lactobacillus plantarum* (SL) alleviates high salinity-induced oxidative stress, lipid metabolism disorders, and ferroptosis in common carp by modulating the PPAR and ferroptosis signaling pathways [11]. Numerous *Bacillus* species possess the ability to reduce selenite into elemental nano-selenium. Among them, *Bacillus licheniformis* has attracted attention for its beneficial roles in aquaculture, including water quality improvement, growth promotion, and immunomodulation [12]. We therefore selected *B. licheniformis* for the bioconversion and production of nano-selenium. Previous research has demonstrated the growth-promoting effects of both *B. licheniformis* and *B. subtilis* on Black Sea roach (*Rutilus frisii kutum*) [13]. Furthermore, although studies on selenium-enriched *B. licheniformis* in aquaculture remain limited, existing evidence shows that selenium-enriched *B. subtilis* can alleviate mercury-induced growth inhibition and inflammation in the mirror carp (*Cyprinus carpio* var. *specularis*) and modulate intestinal microbiota [14]. These findings prompted the present investigation into the potential benefits of selenium-enriched *B. licheniformis* in aquatic feeds.

The intestinal microbiota plays a fundamental role in host health and nutrient metabolism. Selenium supplementation has been demonstrated to significantly influence the composition and abundance of gut microorganisms. Human studies have revealed differences in gut microbiota between populations residing in low- and high-selenium regions [15], while experimental work in mice indicates that moderate selenium intake optimizes gut microbial structure and function [16]. In aquaculture species, nano-selenium has been shown to modulate the gut microbiota of Chinese tongue sole (*Cynoglossus semilaevis*) [17]. Here, we examined the effects of different selenium sources on the intestinal microbiota of largemouth bass, hypothesizing that microbiota modulation may contribute to observed differences in growth performance.

Largemouth bass (*Micropterus salmoides*), native to North America, has gained significant importance in global aquaculture owing to its high market value [18]. Improving the yield and growth efficiency of this species is essential to meet rising consumer demands. The present study was therefore designed to evaluate and compare the effects of different selenium sources—sodium selenite, selenium-enriched *B. licheniformis* fermentation broth (BlSe), and selenium-enriched *S. cerevisiae* fermentation broth (ScSe)—on the growth performance, health status, antioxidant capacity, and intestinal microbiota of largemouth bass.

## Materials and Methods

### Preparation of Selenium-Enriched Fermentation Broth with *Bacillus licheniformis* (BlSe) and *Saccharomyces cerevisiae* (ScSe)

Both *B. licheniformis* and *S. cerevisiae* were provided by Huazhong Agricultural University. The taxonomic classification of the two strains was then identified and *B. licheniformis* was denoted as *B. licheniformis* D1, while *S. cerevisiae* was designated as *S. cerevisiae* JM1. Detailed information of the primers sequences and respective strains can be found in Table S1.

*B. licheniformis* D1 and *S. cerevisiae* JM1 were respectively inoculated in Luria-Bertani (LB) medium at 37°C, 180 rpm for 48 h, and Yeast Extract Peptone Dextrose (YPD) medium at 30°C, 180 rpm for 48 h, both with 0.1 mg/ml of Na_2_SeO_3_ [19]. The completed bacterial liquid is selenium-enriched, probiotic fermentation broth. BlSe is a type of nano-selenium, and ScSe is a type of organic selenium.

Regarding the media, the LB consisted of 10 g/l peptone, 5 g/l yeast extract, and 10 g/l NaCl, while the YPD was composed of 20 g/l peptone, 20 g/l glucose, and 10 g/l yeast paste.

### Analysis of Selenium Composition in BlSe and ScSe

Samples were collected at different times; the first after addition of the strain (as 0 h sample), the second after 48 h of incubation (as 48 h sample). Both obtained samples were centrifuged at 12,000 ×*g* for 10 min. Then, the Se^4+^ concentration of supernatants (0 h and 48 h samples) was quantified using an atomic fluorescence spectrometer, as described by Khoei [20]. The concentration measured for the 0 h sample represented the initial concentration, while that of the 48 h sample was the residual concentration. The transformation of Na_2_SeO_3_ by microorganisms was determined as the difference between the initial sodium selenite concentration and the residual sodium selenite concentration.

### Formulation of Selenium-Enriched Largemouth Bass Feed

Following preparation and formulation of BlSe, ScSe, and Na_2_SeO_3_ (SS) as the selenium sources, they were individually mixed with the feed by spraying to give the feed a selenium content of 1 mg/kg. Theoretically, the selenium content of 1 ml of selenium-enriched bacterial solution was 0.046 mg.

### Selection and Feeding of Largemouth Bass

The Institutional Animal Care and Use Committee of Huazhong Agricultural University approved all experiments (Ethics no: HZAUFI-2025-0036). Three hundred and eighty-four largemouth bass with an initial weight of 9.68 g ± 0.3 g were randomly divided into four groups to include a control and three treatments. The control group (CK) was fed without selenium, and the treatments were fed with SS, BlSe, and ScSe, respectively. Each group had three replicates. Feeding experiments were conducted for eight weeks with daily feedings at 8:00 am and 6:00 pm. The feeding amount was based on the total weight of fish in each tank, and 3% was used in this experiment.

### Sampling of Largemouth Bass upon Completion of Culture

After an 8-week rearing period, fish were collected for comprehensive analysis. Initial measurements were body length and weight. Following the morphometric measurements, blood samples were taken from the caudal vein using a syringe. Subsequently, the specimens were dissected, and samples of muscle tissue, liver tissue, intestines, and intestinal contents were obtained. The intestines were fixed with a 5% paraformaldehyde solution after removing the intestinal contents. The collected muscle tissue, liver tissue, and intestinal contents were preserved in 2-ml, enzyme-free centrifuge tubes, which were promptly placed on dry ice and then stored at -80°C for further analysis. More than four fish were randomly selected per group from each tank, and all samples were triplicates.

### Calculation of Survival Rate (SR), Weight Gain Rate (WGR), Specific Growth Rate (SGR), and Feed Conversion Ratio (FCR)

To investigate the influence of selenium on the production of largemouth bass and its conversion into feed, the SR, WR, SGR and FCR were tested and then calculated using the following formula:

SR = (Final number of survivors / initial number of placements)×100%; WGR = (final weight - initial weight) / initial weight;

SGR = [ln (final weight) - ln (initial weight)] / days of feeding;

FCR = feed consumption / (final weight - initial weight).

### Measurement of Selenium in Muscle Tissue

As the selenium content of muscle tissue is an important criterion for determining whether largemouth bass can be used as selenium-supplemented food, the selenium content was tested with reference to the method proposed by Khoei [20].

### Measurement of Intestinal Mucosal Layer Thickness, Goblet Cells, Villi Length

The thickness of the intestinal mucosal layer, goblet cells, and villi length were determined by sending fixed intestinal tissues (Wuhan Servicebio Technology Co., Ltd., China) for Periodic Acid-Schiff staining (PAS). Subsequently, villi length, mucosal layer thickness, and goblet cells on villous epithelium were analyzed using image analysis software under 40× and 200× magnification microscopes.

### Measurement of Liver Tissue Health

To evaluate the health of liver tissue, serum alanine aminotransferase (ALT), aspartate aminotransferase (AST), and alkaline phosphatase (ALP) in the plasma were measured. The obtained plasma was centrifuged and the serum layer was sent to the Veterinary Research Center of Huazhong Agricultural University. The above three enzymes were assayed with the corresponding kits.

### Measurement of Antioxidant Capacity in Liver Tissue

To analyze the antioxidant capacity of liver tissue, glutathione peroxidase (GSH-Px), malondialdehyde (MDA), and total superoxide dismutase (T-SOD) were tested by assay kit. The liver tissue was turned into a 20% tissue homogenate with 0.7% saline for further analysis. The GSH-Px, MDA, and T-SOD assay kits were produced by Nanjing Jiancheng Institute.

### Intestinal Microbiota Analysis Using 16S rRNA Amplicon

To explore the effects of different selenium sources on the intestinal flora, 16S rRNA gene sequencing was performed. After extracting the intestines, the fat was removed from their surface with phosphate buffer, and the intestinal contents were then extruded with forceps. Subsequently, the contents were sent to Wuhan Benagen Co. for further sequencing analysis. The VAHTS Universal DNA Library Prep Kit for Illumina V3, in conjunction with VAHTS DNA Adapters Set 3 – Set 6 for Illumina (Vazyme, China), were used for library preparation. High-throughput sequencing was performed on the NovaSeq 6000 Sequencer (USA), and 16S rRNA gene-based functional prediction and KEGG pathway analysis using PICRUSt2 functional predictions based on 16S rRNA gene sequencing data were performed using PICRUSt2 (v. 2.5.1). The amplicon sequence variants (ASVs) obtained from QIIME2 (v. 2023.9) were used as input for PICRUSt2.

The analysis pipeline consisted of four main steps: (1) Sequence Placement, in which ASV representative sequences were aligned to a reference phylogenetic tree using HMMER, EPA-NG, and gappa; (2) Hidden-State Prediction, which is used to infer gene family (KO) copy numbers for each ASV based on evolutionary modeling;(3) Metagenome Inference, whereby per-ASV KO predictions were multiplied by ASV abundances to generate per-sample functional profiles; and (4) Pathway Inference, in which KO abundances were mapped to KEGG pathways using the MinPath algorithm to minimize false-positive pathway reconstruction.

The resulting KO and KEGG pathway abundance tables were normalized to relative abundance using total-sum scaling and then categorized into hierarchical KEGG levels (Levels 1–3). Differentially enriched pathways were identified by LEfSe (*p* < 0.05)

### Statistical Analysis

All data were analyzed using GraphPad Prism 8 and one-way ANOVA followed by Tukey’s post-hoc test to determine significant differences among groups (*p* < 0.05).

## Results and Discussion

### Composition of Fermentation Broth with *Bacillus licheniformis* (BlSe) and *Saccharomyces cerevisiae* (ScSe)

**Fig. 1:**
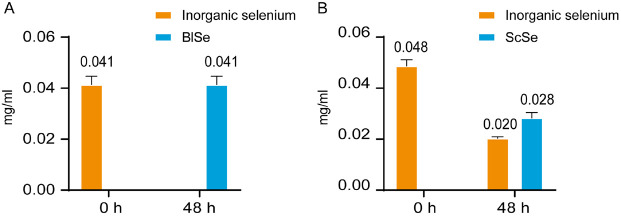
The variation of selenium fractions in BlSe (A), and ScSe (B) at 0 h and 48 h. 0 h is the concentration of Se^4+^ in the medium after accessing the strain, and 48 h is the concentration of Se^4+^ in the medium after completion of incubation.

The selenium compositions of ScSe and BlSe samples at 0 h and 48 h are shown in Fig. 1. The selenite in the BlSe was completely transformed after 48 h (Fig. 1A), while only 60% conversion was observed in ScSe (Fig. 1B). To verify selenium enrichment, the cell pellets obtained from selenium-supplemented cultures displayed a characteristic reddish coloration, indicating the biogenic reduction of selenite to elemental selenium in Fig. S1. The precise mechanism behind this discrepancy in selenite conversion efficiency remains elusive. However, previous studies on the efficiency of selenite conversion by strains of the same species have shown that *S. cerevisiae* can convert 55-60% of selenite at 1-10 μg/ml of selenite [21], whereas *B. licheniformis* demonstrates a conversion efficiency of 97%at 500 μg/ml [22]. In addition, studies on selenite tolerance revealed that *B. licheniformis* exhibits greater tolerance compared to *S. cerevisiae* [22, 23]. Despite both strains displaying similar performance in this study, we hypothesized that the variation in conversion efficiency is linked to their differential selenite tolerance capabilities.

### Effects of Different Selenium Sources on Largemouth Bass

To investigate the effects of selenium sources on the growth of largemouth bass, a dosage of 1 mg Se/kg was systematically added to the feed. As delineated in Table 1, fish mortality occurred in the SS group, which was presumably related to the toxicity of sodium selenite [24]. The ScSe group showed a significant increase in both weight gain rate (WGR) and specific growth rate (SGR) in comparison with the CK group. This finding is similar with the study of Nile tilapia (*Oreochromis niloticus*) that yeast selenium promotes growth more effectively [25]. Enhanced feed utilization significantly contributes to the accelerated growth rate observed in fish.

### Effects of Different Selenium Sources on the Selenium Content in Largemouth Bass Muscle Tissue

**Fig. 2:**
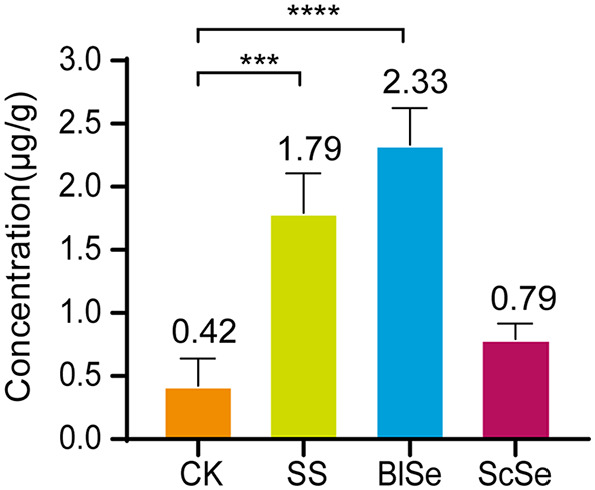
Muscle tissues selenium content in CK, SS, BlSe, and ScSe groups. The symbol "***" presents *P* < 0.001 , and "****" stands out *P* < 0.0001. Both of them are statistically different.

Fish is a major part of our diet, and selenium levels in muscle tissue can be used as a way to assess its ability to provide us with selenium [26]. As shown in Fig. 2, the selenium content exhibited an increase across all treatments in comparison to the CK. Notably, the increase of selenium content in the ScSe group was comparatively less apparent, whereas the SS and BlSe groups showed statistically significant increase. The phenomenon also existed in juvenile grass carp, where it was shown that selenium supplementation contributes to heightened selenium content in the muscles and increased protein levels, as reported by Wang *et al*. [27]. The ScSe group exhibited only a smaller increase in muscle selenium content, which led to the discovery of the smallest feed coefficient in this group. This indicates that the ScSe group gained the most weight when consuming the same amount of feed. Moreover, when considering feed of equal quality containing a specific amount of selenium, a higher weight gain corresponded to lower muscle selenium content.

### Effects of Different Selenium Sources on the Intestinal Tract in Largemouth Bass

**Fig. 3:**
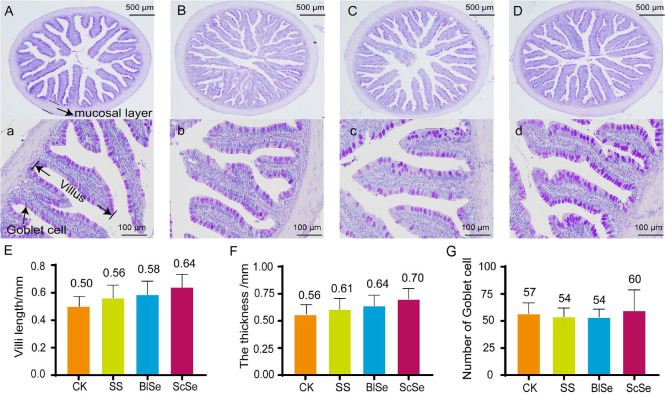
The effect of SS, BlSe and ScSe on the intestinal tract in largemouth bass. **A-D** are PAS graphs of CK , SS, BlSe, and ScSe under 40× magnification microscopes. **a-d** are PAS graphs of CK, SS, PAS BlSe, and ScSe under 200× magnification microscopes. E is the length of villi in CK, SS, BlSe, and ScSe groups, F is the thickness of the mucosal layer, and G is the number of goblet cells per unit length.

The intestinal tract facilitates the digestive process in animals. The morphological characteristics (Fig. 3A-3D under 40× magnification microscopes, Fig. 3a-3d under 200× magnification microscopes) of intestinal villi length and the thickness of the mucous membrane layer can be an indicator of digestive health in largemouth bass, and the abundance of goblet cells can reflect the immunity of animals [28]. As shown in Fig. 3E-3G, three treatments revealed an increase in villi length and thickness of the mucosal layer, but there was no significant impact on the abundance of goblet cells. According to these results, selenium might have the ability to improve intestinal digestibility of largemouth bass. Previous studies also mentioned that selenium could increase villi length and the abundance of goblet cells in aquatic animals, specifically Nile tilapia [29]. This suggests that three selenium sources will have some positive effect on digestive ability and health of the intestinal tract.

### Effects of Different Sources on the Serum Levels of ALT, AST and ALP in Largemouth Bass

**Fig. 4:**
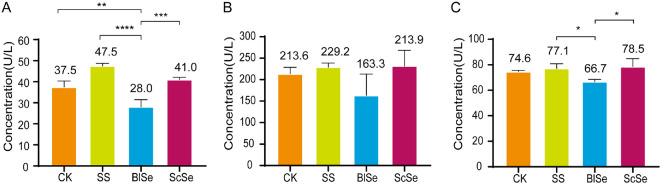
The effects of SS, BlSe, ScSe on the enzymes in the serum of largemouth bass. A shows ALT concentration of CK, SS, BlSe, ScSe, B shows the AST concentration, C shows ALP concentration. The symbol "*" presents *P* < 0.05, "**" presents *P* < 0.01, "***" presents *P* < 0.001, and "****" stands out *P* < 0. 0001. Both are statistically different.

The serum levels of ALT, AST and ALP are indicative of hepatic health, and an increase in their content indicates that the liver has been damaged to a certain extent [30]. As shown in Fig. 4A, compared to the CK group, ALT levels increased by 27% in the SS group, decreased by 25% in the BlSe group, and increased by 9% in the ScSe group. As shown in Fig. 4B, AST levels increased by 7% in the SS group, decreased by 24% in the BlSe group, and increased by 9% in the ScSe group. As shown in Fig. 4C, ALP levels increased by 3% in the SS group, decreased by 10% in the BlSe group, and increased by 5% in the ScSe group. The levels of ALT, AST, and ALP were lower in the BlSe group than in the CK group, whereas their levels were higher in the SS and ScSe groups than in the CK group. This suggests that BlSe showed mild hepatoprotective effects, as evidenced by moderate decreases in these enzymes compared with the control group. Also selenium and vitamin C can effectively diminish serum levels of ALT, AST, and ALP, thereby mitigating liver damage caused by substances such as fenitrothion in rats [31]. Meanwhile, regarding the role of *B. licheniformis*, studies on this bacterium’s effect on the growth performance of weaned piglets have shown that it can alleviate and reduce their inflammatory factors [32, 33]. The two studies mentioned above showed that both selenium and *B. licheniformis* had a mitigating effect on hepatic inflammation in animals. This is consistent with the alleviation by BlSe of hepatic inflammation in largemouth bass as shown in the present study.

### Effects of Different Selenium Sources on the Antioxidant Capacity of Liver Tissues in Largemouth Bass

**Fig. 5:**
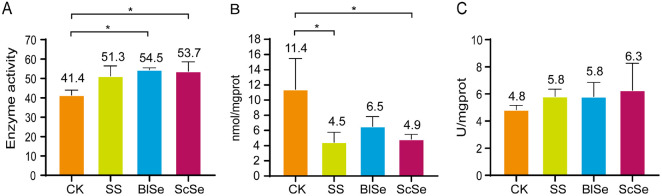
The effects of SS, BlSe, ScSe on the antioxidant capacity in largemouth bass. A shows the GSH-Px content, B shows the MDA content, and C stands for the T-SOD content. "*" presents *P* < 0.05 with a statistical difference.

The liver plays a crucial role in the body's overall ability to fend off oxidative damage, which is key for maintaining good health, as described in detail by Li, *et al*. [34]. One important aspect is the presence of selenocysteine, which acts as the active center of an enzyme called glutathione peroxidase (GSH-Px). The level of selenium in the body is closely linked to the activity of GSH-Px [35]. GSH-Px is vital for combating oxidative stress as it protects cell membranes and neutralizes harmful peroxides. As shown in Fig. 5A, a significant increase in GSH-Px activities in the liver tissues was observed across all treatments compared to the CK group. It indicates that each treatment effectively enhances the antioxidant capacity of largemouth bass. Furthermore, the heightened activities of GSH-Px indicate a strengthened defense against oxidative stress, underlining the positive impact of these treatments on the overall antioxidant capabilities of largemouth bass.

In organisms, the oxidation of free radicals and lipids produces malondialdehyde (MDA), which causes cross-linking of essential macromolecules, such as proteins and nucleic acids. MDA is cytotoxic and a class 3 carcinogen. MDA interferes with the functioning of mitochondrial respiratory chain complexes and key enzymes within the mitochondrion. Additionally, it also exacerbates membrane damage, thus the amount of MDA present reflects both the extent of lipid peroxidation and the inherent antioxidant prowess within the organism [36]. As shown in Fig. 5B, the MDA contents pertaining to the various treatments exhibited a statistically significant reduction in comparison to the CK group. This reduction aligns with the measurements of GSH-Px, further demonstrating that selenium can effectively enhance the antioxidant capacity of largemouth bass. The decrease in MDA indicates a decrease in lipid peroxidation and suggests an improvement in the organism’s ability to combat oxidative stress, which is consistent with the increased activity of GSH-Px observed in the previous analysis.

Superoxide dismutase (SOD) exerts antioxidant effects through the enzymatic facilitation of superoxide anion radicals into hydrogen peroxide (H_2_O_2_) and molecular oxygen (O_2_), which is recognized as one of the most efficient free radical scavengers within the biological system [37]. As shown in Fig. 5C, there was an enhancement in the treatments compared to the CK group. In addition, all three selenium sources showed an increase in the hepatic antioxidant capacity of largemouth bass

Selenium has always played an important role in antioxidant function, and the present experiment demonstrated that selenium significantly enhanced the antioxidant capacity of the liver of largemouth bass. Similarly, it also has performed excellently in other aquatic animals. For example, selenium can increase GSH-Px, SOD activity, and reduce MDA content in shrimp [38]. Additionally, it has been shown in fish that selenium increases GSH-Px activity and decreases MDA content in juvenile Black Sea bream (*Acanthopagrus schlegelii*) [39]. The above indicates that selenium is an excellent antioxidant for application in aquaculture.

### Effects of Different Selenium Sources on the Intestinal Microbiota of Largemouth Bass

**Fig. 6:**
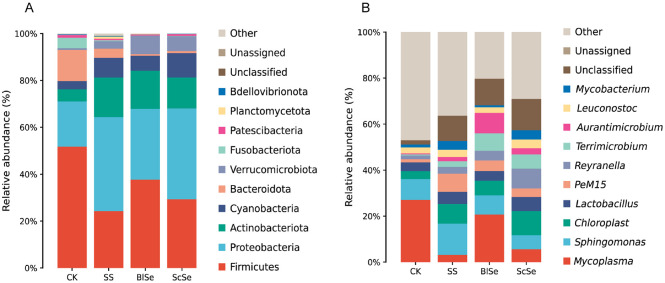
The effects of SS, BlSe, ScSe on the intestinal flora in largemouth bass. **A** is the abundance of flora at the phylum level, **B** is the abundance of flora at the genus level.

The gut serves as a pivotal locus for the intricate processes of digestion in the realm of digestive physiology. The gut microorganisms that attract more attention due to abnormalities in gut health are likely to be related to overall bodily health [40]. Differential analyses of gut microbiota were performed at both the phylum and genus levels. As shown in Fig. 6A, there were four major phyla, Bacteroidetes, Firmicutes, Proteobacteria, and Actinobacteria in the various treatments. In comparison to CK group, a decrease in the abundance of Firmicutes and Bacteroidetes was noted, accompanied by an increase in the abundance of Proteobacteria and Actinobacteria within the treatments. Moreover, Verrucomicrobia was the only phylum with a significant difference and had the highest abundance in the BlSe group. Detailed abundance of Verrucomicrobia in each group and differential analyses of its abundance are shown in Fig. S2A. At the genus level, as shown in Fig. 6B, the abundance of *Mycoplasma* decreased in the treatments compared to the group of CK, with the BlSe group showing a comparatively milder reduction, while the SS and ScSe groups exhibited more pronounced decreases. In contrast to the other three groups, BlSe demonstrated higher levels of *Aurantimicrobium* and *Terrimicrobium*, with the abundance of *Terrimicrobium* significantly increased as shown in Fig. S2B. Meanwhile, *Reyranella* in the ScSe group showed elevated levels compared to other groups, demonstrating a significant increase compared to the CK group, as shown in Fig. S2C. *Sphingomonas* displayed relatively high abundance in the SS group. Further analyses of abundance variability for other phyla and genera are presented in Fig. S3.

The elevation of Verrucomicrobia was concurrent with a rise in *Terrimicrobium*, which belong to Verrucomicrobiota [41]. It has been previously shown that among freshwater fish, the major intestinal microbiota of carp consists of Proteobacteria, Firmicutes, Fusobacteria, and Bacteroidetes [17]. We also found higher abundance of Firmicutes, Proteobacteria, Bacteroidetes in all groups, but Fusobacteria had higher abundance only in the CK group, which was not significantly observed in the other treatments. Also, higher abundances of Cyanobacteria and Actinobacteria were present in all groups. In a review, Kakakhel *et al*. hypothesized that a decrease in Actinobacteria may affect fish immunity, leading to disease [42]. Therefore, the increase in the abundance of Actinobacteria in all treatments has the potential to enhance the immunity of largemouth bass. Many studies of *Terrimicrobium* and *Reyranella* only focused on screening efforts, while few studies have examined the effects of increased or decreased abundance of either in the gut microbial community. However, in a study of arsenic on the carp gut, it was stated that arsenic decreased the abundance of the beneficial bacterium *Reyranella* [43]. In this case, the abundance of *Reyranella* was upregulated in all treatments, which might have a positive effect on the intestinal microbiota based on the study of the carp gut. Different selenium sources might impact the gut microbial communities, and thus may result in the difference of the growth, muscle selenium content, and the liver tissue of largemouth bass. However, whether selenium has a beneficial or detrimental effect on intestinal microbiota of largemouth bass needs further study.

### Effects of Different Selenium Sources on the Physiological Functions of Largemouth Bass

**Fig. 7:**
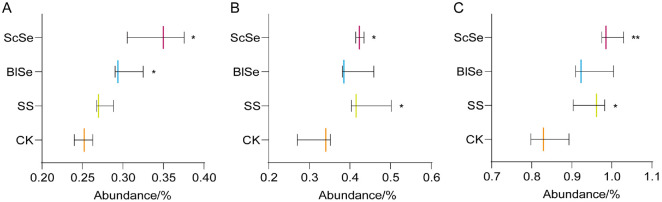
The effects of SS, BlSe, ScSe on the tertiary functional composition analysis of largemouth bass A is the abundance and difference of taurine and hypotaurine metabolism in CK, SS, BlSe, ScSe; B is the abundance and difference of biosynthesis of unsaturated fatty acids in CK, SS, BlSe, ScSe; C is the abundance and difference of fatty acid biosynthesis in CK, SS, BlSe, ScSe. Symbols: "*" presents *P* < 0.05, "**" presents *P* < 0.01 statistically different.

To predict sample functional abundance based on marker gene sequence abundance in the sample, PICRUSt2 software was used [44]. A KEGG functional composition was performed, revealing no significant differences in the secondary functional composition among groups, as shown in Fig. S4. Consequently, a subsequent analysis targeted the tertiary metabolic pathways, as shown in Fig. S5. Meanwhile, Fig. 7 illustrates three pathways, exhibiting notable distinctions in KEGG tertiary functional analysis. In Fig. 7A, both BlSe and ScSe exhibited significant elevations compared to CK, while SS demonstrated an increase, though not reaching statistical significance in taurine and hypotaurine taurine metabolism. As shown in Fig. 7B, biosynthesis of unsaturated fatty acids displayed elevated levels in SS, BlSe, and ScSe compared to CK, with SS and ScSe achieving statistical significance. As shown in Fig. 7C, ScSe and SS were significantly elevated compared to CK, while BlSe was less elevated in fatty acid biosynthesis.

Taurine and hypotaurine metabolism is associated with cysteine [45], and prompted the hypothesis that selenium supplementation could stimulate cysteine synthesis. This conjecture is grounded in the established role of selenocysteine as an important constituent of selenoenzymes [46]. Previous KEGG analyses on maize seedlings demonstrated that low selenium concentrations upregulate glutathione (GSH) metabolism [47]. It is hypothesized that selenium may promote biological synthesis of GSH. The upregulation of the abundance of two metabolic pathways regarding fatty acid synthesis is consistent with the upregulation of the abundance of lipid synthesis in the secondary functional composition, but no other studies with similar results were found.

Although this study primarily focused on selenium-enriched probiotics to reflect their practical application, the absence of non-enriched Bl and Sc controls may confound the interpretation of selenium-specific effects. Future studies will include non-enriched strains to further confirm the role of selenium enrichment. In addition, although this study used a single supplementation level to evaluate the physiological effects of selenium over an 8-week period, further research with multiple concentrations and longer feeding durations is warranted to determine optimal dosages and assess long-term safety for practical aquaculture applications.

## Conclusion

This study systematically compared the physiological responses of largemouth bass (*Micropterus salmoides*) to different selenium sources used as dietary supplements. Selenium-enriched *S. cerevisiae* fermentation broth (ScSe) significantly improved weight gain rate and specific growth rate, whereas selenium-enriched *B. licheniformis* fermentation broth (BlSe) markedly enhanced hepatic health, as reflected by reduced serum levels of alanine aminotransferase (ALT), aspartate aminotransferase (AST), and alkaline phosphatase (ALP). All selenium forms consistently strengthened the antioxidant capacity in liver tissue, demonstrated by elevated activities of glutathione peroxidase (GSH-Px) and total superoxide dismutase (T-SOD), along with decreased malondialdehyde (MDA) content. Moreover, selenium supplementation regulated intestinal microbiota, inducing shifts at both phylum and genus levels. KEGG pathway analysis further indicated that selenium treatments influenced several biological functions, particularly those involved in antioxidant and lipid metabolism. Notably, BlSe exhibits strong potential for commercial application, not only due to its efficacy in promoting growth and health but also owing to its practical advantages in production. Specifically, *B. licheniformis* demonstrates high efficiency in converting inorganic selenium into bioactive organic or nano forms, and its spore-forming ability confers enhanced environmental stress resistance, thereby improving the stability and shelf-life of the feed additive. In conclusion, both ScSe and BlSe represent effective selenium sources for aquaculture, with BlSe offering distinct benefits in terms of manufacturing scalability and functional performance.

## Supplemental Materials

Supplementary data for this paper are available on-line only at http://jmb.or.kr.



## Figures and Tables

**Table 1:** The weight gain rate, specific growth rate and feed conversion ratio in CK, SS, BlSe, ScSe groups. Asterisks present "*p* < 0.05" with a statistical difference.

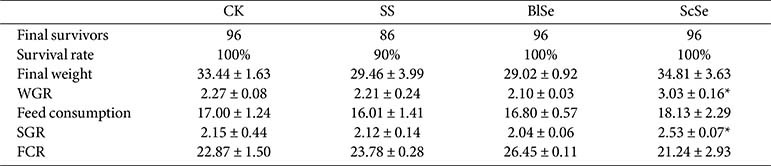

## References

[1] Bell JG, Cowey CB (1989). Digestibility and bioavailability of dietary selenium from fishmeal, selenite, selenomethionine and selenocystine in Atlantic salmon (Salmo salar). Aquaculture.

[2] Zheng Y, Xie T, Li S, Wang W, Wang Y, Cao Z (2021). Effects of selenium as a dietary source on performance, inflammation, cell damage, and reproduction of livestock induced by heat stress: a review. Front. Immunol..

[3] Dinh QT, Cui Z, Huang J, Tran TAT, Wang D, Yang W (2018). Selenium distribution in the Chinese environment and its relationship with human health: A review. Environ. Int..

[4] Dawood MAO, Koshio S, Zaineldin AI, Van Doan H, Ahmed HA, Elsabagh M (2019). An evaluation of dietary selenium nanoparticles for red sea bream (Pagrus major) aquaculture: growth, tissue bioaccumulation, and antioxidative responses. Environ. Sci. Pollut. Res. Int..

[5] Hamilton SJ (2004). Review of selenium toxicity in the aquatic food chain. Sci. Total Environ..

[6] Vijayaram S, Ghafarifarsani H, Vuppala S, Nedaei S, Mahendran K, Murugappan R (2025). Selenium nanoparticles: revolutionizing nutrient enhancement in aquaculture - a review. Biol. Trace Elem. Res..

[7] Xing Y, Wang P, Wen S, Zhang M, Gisbert E, Todorcevic M (2025). Protective effect of selenium supplementation against damage of health and muscle quality in *Onychostoma macrolepis* under a thermally oxidized fish oil-enriched diet. Fish Physiol. Biochem..

[8] Hou X, Wang Z, Peng M (2025). Selenium compounds and their bioactivities: molecular mechanisms and prospects for functional food and therapeutic applications. Plants.

[9] Fonseca SBd, Silva JHVd, Filho EMB, Mendes PdP, Fernandes JBK, Amâncio ALdL, *et al.* 2013. Influence of levels and forms of selenium associated with levels of vitamins C and E on the performance, yield and composition of tilapia fillet. *Food Sci. Technol. Int.* **33:** 109-115. 10.1590/S0101-20612013000500017

[10] Wang C, Lovell RT, Klesius PH (1997). Response to *Edwardsiella ictaluri* challenge by channel catfish fed organic and inorganic sources of selenium. J. Aquatic Anim. Health.

[11] Shang X, Che X, Geng L, Zhang Q, Wei H, Li W (2025). Transcriptomic analysis reveals that selenium-enriched *Lactobacillus plantarum* alleviates high -salinity stress in common carp through lipid metabolism and ferroptosis signalling pathways. Ecotoxicol. Environ. Saf..

[12] James G, Das BC, Jose S, V.J RK. 2021. *Bacillus* as an aquaculture friendly microbe. *Aquac. Int.* **29:** 323-353. 10.1007/s10499-020-00630-0

[13] Azarin H, Aramli MS, Imanpour MR, Rajabpour M (2015). Effect of a probiotic containing *Bacillus licheniformis* and *Bacillus subtilis* and ferroin solution on growth performance, body composition and haematological parameters in kutum (*Rutilus frisii kutum*) fry. Probiotics Antimicrob. Proteins.

[14] Shang X, Wang B, Sun Q, Zhang Y, Lu Y, Liu S (2022). Selenium-enriched *Bacillus subtilis* reduces the effects of mercury-induced on inflammation and intestinal microbes in carp (*Cyprinus carpio* var. specularis). Fish Physiol. Biochem..

[15] Zhang ZX, Xiang H, Sun GG, Yang YH, Chen C, Li T (2021). Effect of dietary selenium intake on gut microbiota in older population in Enshi region. Genes Environ..

[16] Zhai Q, Cen S, Li P, Tian F, Zhao J, Zhang H (2018). Effects of dietary selenium supplementation on intestinal barrier and immune responses associated with its modulation of gut microbiota. Environ. Sci. Technol. Lett..

[17] Jia L, Chen C, Zhao N, He X, Zhang B (2022). Effects of low and high levels of nano-selenium on intestinal microbiota of Chinese tongue sole (*Cynoglossus semilaevis*). Aquac. Fish..

[18] Fantini LE, Smith MA, Jones M, Roy LA, Lochmann R, Kelly AM (2021). Growth parameters in northern largemouth bass *Micropterus salmoides* salmoides raised near their upper thermal tolerance for 28 days. Aquac. Rep..

[19] Wang Q, Wang C, Kuang S, Wang D, Shi Y (2023). Biological selenite reduction, characterization and bioactivities of selenium nanoparticles biosynthesised by *Pediococcus acidilactici* DSM20284. Molecules.

[20] Khoei NS, Lampis S, Zonaro E, Yrjala K, Bernardi P, Vallini G (2017). Insights into selenite reduction and biogenesis of elemental selenium nanoparticles by two environmental isolates of *Burkholderia fungorum*. N. Biotechnol..

[21] Sánchez-Martínez M, da Silva EGP, Pérez-Corona T, Cámara C, Ferreira SLC, Madrid Y (2012). Selenite biotransformation during brewing. Evaluation by HPLC-ICP-MS. Talanta.

[22] Ikram M, Faisal M (2010). Comparative assessment of selenite (SeIV) detoxification to elemental selenium (Se0) by *Bacillus* sp. Biotechnol. Lett..

[23] Golubev VI, Golubev NV (2002). Selenium Tolerance of Yeasts. Microbiology.

[24] Wang L, Sagada G, Wang R, Li P, Xu B, Zhang C (2022). Different forms of selenium supplementation in fish feed: the bioavailability, nutritional functions, and potential toxicity. Aquaculture.

[25] Chen H, Li J, Yan L, Cao J, Li D, Huang G-Y (2020). Subchronic effects of dietary selenium yeast and selenite on growth performance and the immune and antioxidant systems in Nile tilapia *Oreochromis niloticus*. Fish Shellfish Immunol..

[26] Yamashita Y, Yamashita M, Iida H (2013). Selenium content in seafood in Japan. Nutrients.

[27] Wang Y, Yan X, Fu L (2013). Effect of selenium nanoparticles with different sizes in primary cultured intestinal epithelial cells of crucian carp, *Carassius auratus gibelio*. Int. J. Nanomed..

[28] Huo X, Zhang Q, Chang J, Yang G, He S, Yang C (2023). Nanopeptide C-I20 as a novel feed additive effectively alleviates detrimental impacts of soybean meal on mandarin fish by improving the intestinal mucosal barrier. Front. Immunol..

[29] Ghazi S, Diab AM, Khalafalla MM, Mohamed RA (2022). Synergistic effects of selenium and zinc oxide nanoparticles on growth performance, hemato-biochemical profile, immune and oxidative stress responses, and intestinal morphometry of nile tilapia (*Oreochromis niloticus*). Biol. Trace Element Res..

[30] Lohner TW, Reash RJ, Willet VE, Fletcher J (2001). Assessment of tolerant sunfish populations (*Lepomis* sp.) inhabiting seleniumladen coal ash effluents. 3. Serum chemistry and fish health indicators. Ecotoxicol. Environ. Saf..

[31] Milošević MD, Paunović MG, Matić MM, Ognjanović BI, Saičić ZS (2018). Role of selenium and vitamin C in mitigating oxidative stress induced by fenitrothion in rat liver. Biomed Pharmacother..

[32] Yu X, Cui Z, Qin S, Zhang R, Wu Y, Liu J (2022). Effects of *Bacillus licheniformis* on growth performance, diarrhea incidence, antioxidant capacity, immune function, and fecal microflora in weaned piglets. Animals (Basel).

[33] Yu X, Dai Z, Cao G, Cui Z, Zhang R, Xu Y (2023). Protective effects of *Bacillus licheniformis* on growth performance, gut barrier functions, immunity and serum metabolome in lipopolysaccharide-challenged weaned piglets. Front. Immunol..

[34] Li S, Li H, Xu X, Saw PE, Zhang L (2020). Nanocarrier-mediated antioxidant delivery for liver diseases. Theranostics.

[35] Shini S, Sultan A, Bryden W (2015). Selenium biochemistry and bioavailability: implications for animal agriculture. Agriculture.

[36] Yuan R, Tao X, Liang S, Pan Y, He L, Sun J (2018). Protective effect of acidic polysaccharide from *Schisandra chinensis* on acute ethanol-induced liver injury through reducing CYP2E1-dependent oxidative stress. Biomed Pharmacother..

[37] Wang F, Liu JC, Zhou RJ, Zhao X, Liu M, Ye H (2017). Apigenin protects against alcohol-induced liver injury in mice by regulating hepatic CYP2E1-mediated oxidative stress and PPARα-mediated lipogenic gene expression. Chem. Biol. Interact..

[38] Kong Y, Ding Z, Zhang Y, Ye J, Du Z (2017). Dietary selenium requirement of juvenile oriental river prawn *Macrobrachium nipponense*. Aquaculture.

[39] Wang L, Xiao JX, Hua Y, Xiang XW, Zhou YF, Ye L (2019). Effects of dietary selenium polysaccharide on growth performance, oxidative stress and tissue selenium accumulation of juvenile black sea bream, *Acanthopagrus schlegelii*. Aquaculture.

[40] de Vos WM, Tilg H, Van Hul M, Cani PD (2022). Gut microbiome and health: mechanistic insights. Gut.

[41] Qiu YL, Kuang XZ, Shi XS, Yuan XZ, Guo RB. 2014. *Terrimicrobium sacchariphilum* gen. nov., sp. nov., an anaerobic bacterium of the class 'Spartobacteria' in the phylum *Verrucomicrobia*, isolated from a rice paddy field. *Int. J. Syst. Evol. Microbiol.* **64:** 1718-1723. 10.1099/ijs.0.060244-0 24535138

[42] Kakakhel MA, Narwal N, Kataria N, Johari SA, Zaheer Ud Din S, Jiang Z (2023). Deciphering the dysbiosis caused in the fish microbiota by emerging contaminants and its mitigation strategies-A review. Environ. Res..

[43] Shi X, Xu W, Che X, Cui J, Shang X, Teng X (2023). Effect of arsenic stress on the intestinal structural integrity and intestinal flora abundance of *Cyprinus carpio*. Front. Microbiol..

[44] Douglas GM, Maffei VJ, Zaneveld JR, Yurgel SN, Brown JR, Taylor CM (2020). PICRUSt2 for prediction of metagenome functions. Nat. Biotechnol..

[45] Sapse AM. 2000. Taurine and Hypotaurine. *In*: Sapse AM (ed.), *Molecular Orbital Calculations for Amino Acids and Peptides*, Birkhäuser Boston, Boston, MA, pp. 74-82. 10.1007/978-1-4612-1354-3_7

[46] Böck A, Forchhammer K, Heider J, Leinfelder W, Sawers G, Veprek B (1991). Selenocysteine: the 21st amino acid. Mol. Microbiol..

[47] Dou L, Tian Z, Zhao Q, Xu M, Zhu Y, Luo X (2021). Transcriptomic characterization of the effects of selenium on maize seedling growth. Front. Plant Sci..

