# Supplementary material for: Biosynthetic Nano-Selenium by Bacillus licheniformis Enhances Growth and Health of Largemouth Bass (Micropterus salmoides)
Source: J Microbiol Biotechnol. 2025 Dec 19;35:e2508049. doi: 10.4014/jmb.2508.08049 (PMC12740848; doi:10.4014/jmb.2508.08049)
Supplement: Supplementary file 1 [file jmb-35-e2508049-supple.pdf]

J. Microbiol. Biotechnol. 2025. 35: e2508049

https://doi.org/10.4014/jmb.2508.08049

Supplementary Table and Figures

Biosynthetic Nano-Selenium by *Bacillus licheniformis* Enhances Growth and Health of Largemouth Bass (*Micropterus salmoides*)

Xiaohu Li[1], Ge Bai[1], Lifei Feng[2], Honghao Ding[1], Keke Li[2], Wen Zhou[3], Yunxiang Liang[1], Yingjun Li[1*]

[1]National Key Laboratory of Agricultural Microbiology, College of Life Science and Technology, Huazhong Agricultural University, Wuhan 430070, China

[2]Henan Jinbaihe Biotechnology Co., Ltd., Tangyin, Anyang 455000, China

[3]State Key Laboratory of Green Pesticide, Central China Normal University, Wuhan 430079, China

*Corresponding author: yingjun@mail.hzau.edu.cn

Table S1. Information on strain identification. The following sequences are primer sequences, 16S rDNA sequence *of Bacillus licheniformis* D1 and 18S rDNA sequence of *Saccharomyces cerevisiae* JM1.

Prime 27 F sequence	5'-AGAGTTTGATCCTGGCTCAG-3'
Prime 1492 R sequence	5'-GGTTACCTTGTTACGACTT-3'
Prime EUK A sequence	5'-AACCTGGTTGATCCTGCCAGT-3'
Prime EUK B sequence	5'-TGATCCTTCTGCAGGTTCACCTAC-3'
Baclillus licheniformis D1,16S rDNA sequence	AAGGTTACCTCACCGACTTCGGGTGTTACAAACTCTCGTGGTGTGACGGG<br>CGGTGTGTACAAGGCCCGGGAACGTATTCACCGCGGCATGCTGATCCGCG<br>ATTACTAGCGATTCCAGCTTCACGCAGTCGAGTTGCAGACTGCGATCCGA<br>ACTGAGAACAGATTTGTGGGATTGGCTTAGCCTCGCGGCTTCGCTGCCCT<br>TTGTTCTGCCCATTGTAGCACGTGTGTAGCCCAGGTCATAAGGGGCATGAT<br>GATTTGACGTCATCCCCACCTTCCTCCGGTTTGTCACCGGCAGTCACCTTA<br>GAGTGCCCAACTGAATGCTGGCAACTAAGATCAAGGGTTGCGCTCGTTGC<br>GGGACTTAACCCAACATCTCACGACACGAGCTGACGACAACCATGCACC<br>ACCTGTCACTCTGCCCCCGAAGGGGAAGCCCTATCTCTAGGGTTGTCAGA<br>GGATGTCAAGACCTGGTAAGGTTCTTCGCGTTGCTTCGAATTAAACCACA<br>TGCTCCACCGCTTGTGCGGGCCCCCGTCAATTCCTTTGAGTTTCAGTCTTG<br>CGACCGTACTCCCCAGGCGGAGTGCTTAATGCGTTTGCTGCAGCACTAAA<br>GGGCGGAAACCCTCTAACACTTAGCACTCATCGTTTACGGCGTGGACTAC<br>CAGGGTATCTAATCCTGTTCGCTCCCCACGCTTTCGCGCCTCAGCGTCAGT<br>TACAGACCAGAGAGTCGCCTTCGCCACTGGTGTTCCTCCACATCTCTACG<br>CATTTCACCGCTACACGTGGAATTCCACTCTCCTCTTCTGCACTCAAGTTC<br>CCCAGTTTCCAATGACCCTCCCCGGTTGAGCCGGGGGCTTTCACATCAGA<br>CTTAAGAAACCGCCTGCGCGCGCTTTACGCCCAATAATTCCGGACAACGC<br>TTGCCACCTACGTATTACCGCGGCTGCTGGCACGTAGTTAGCCGTGGCTTT<br>CTGGTTAGGTACCGTCAAGGTACCGCCCTATTCGAACGGTACTTGTTCTTC<br>CCTAACAACAGAGTTTTACGATCCGAAAACCTTCATCACTCACGCGGCGT<br>TGCTCCGTCAGACTTTCGTCCATTGCGGAAGATTCCCTACTGCTGCCTCCC<br>GTAGGAGTCTGGGCCGTGTCTCAGTCCCAGTGTGGCCGATCACCCTCTCA<br>GGTCGGCTACGCATCGTTGCCTTGGTGAGCCGTTACCTCACCAACTAGCT<br>AATGCGCCGCGGGTCCATCTGTAAGTGGTAGCTAAAAGCCACCTTTTATAA<br>TTGAACCATGCGGTTCAATCAAGCATCCGGTATTAGCCCCGGTTTCCCGGA<br>GTTATCCCAGTCTTACAGGCAGGTTACCCACGTGTTACTCACCCGTCCGCC<br>GCTAACATCAGGGAGCAAGCTCCCATCTGTCCGCTCGACTTGCATGTATTA<br>GGCACGCCGCCAGCGTTCGTCCTGA
Saccharomyces cerevisiae JM1,18S rDNA	CATGTCTAAGTATAAGCAATTTATACAGTGAAACTGCGAATGGCTCATTAA<br>ATCAGTTATCGTTTATTTGATAGTTCCTTTACTACATGGTATAACTGTGGTAA<br>TTCTAGAGCTAATACATGCTTAAAATCTCGACCCTTTGGAAGAGATGTATT

sequence	TATTAGATAAAAAATCAATGTCTTCGGACTCTTTGATGATTCATAATAACTT<br>TTCGAATCGCATGGCCTTGTGCTGGCGATGGTTCATTCAAATTTCTGCCCT<br>ATCAACTTTCGATGGTAGGATAGTGGCCTACCATGGTTTCAACGGGTAACG<br>GGGAATAAGGGTTCGATTCCGGAGAGGGAGCCTGAGAAACGGCTACCAC<br>ATCCAAGGAAGGCAGCAGGCGCGCAAATTACCCAATCCTAATTCAGGGAG<br>GTAGTGACAATAAATAACGATACAGGGCCCATTCGGGTCTTGTAATTGGAA<br>TGAGTACAATGTAAATACCTTAACGAGGAACAATTGGAGGGCAAGTCTGG<br>TGCCAGCAGCCGCGGTAATTCCAGCTCCAATAGCGTATATTAAAGTTGTTG<br>CAGTTAAAAAGCTCGTAGTTGAACTTTGGGCCCGGTTGGCCGGTCCGATT<br>TTTTCGTGTACTGGATTTCCAACGGGGCCTTTCCTTCTGGCTAACCTTGAG<br>TCCTTGTGGCTCTTGGCGAACCAGGACTTTTACTTTGAAAAAATTAGAGT<br>GTTCAAAGCAGGCGTATTGCTCGAATATATTAGCATGGAATAATAGAATAG<br>GACGTTTGGTTCTATTTTGTTGGTTTCTAGGACCATCGTAATGATTAATAGG<br>GACGGTCGGGGGCATCAGTATTCAATTGTCAGAGGTGAAATTCTTGGATT<br>TATTGAAGACTAACTACTGCGAAAGCATTTGCCAAGGACGTTTTCATTAAT<br>CAAGAACGAAAGTTAGGGGATCGAAGATGATCAGATACCGTCGTAGTCTT<br>AACCATAAACTATGCCGACTAGGGATCGGGTGGTGTTTTTTTAATGACCCA<br>CTCGGCACCTTACGAGAAATCAAAGTCTTTGGGTTCTGGGGGGAGTATGG<br>TCGCAAGGCTGAAACTTAAAGGAATTGACGGAAGGGCACCACCAGGAGT<br>GGAGCCTGCGGCTTAATTTGACTCAACACGGGGAAACTCACCAGGTCCA<br>GACACAATAAGGATTGACAGATTGAGAGCTCTTTCTTGATTTTGTGGGTG<br>GTGGTGCATGGCCGTTCTTAGTTGGTGGAGTGATTTGTCTGCTTAATTGCG<br>ATAACGAACGAGACCTTAACCTACTAAATAGTGGTGCTAGCATTTGCTGGT<br>TATCCACTTCTTAGAGGGACTATCGGTTTCAAGCCGATGGAAGTTTGAGG<br>CAATAACAGGTCTGTGATGCCCTTAGACGTTCTGGGCCGCACGCGCGCTA<br>CACTGACGGAGCCAGCGAGTCTAACCTTGGCCGAGAGGTCTTGGTAATCT<br>TGTGAAACTCCGTCGTGCTGGGGATAGAGCATTGTAATTATTGCTCTTCAA<br>CGAGGAATTCCTAGTAAGCGCAAGTCATCAGCTTGCGTTGATTACGTCCCT<br>GCCCTTTGTACACACCGCCCGTCGCTAGTACCGATTGAATGGCTTAGTGAG<br>GCCTCAGGATCTGCTTAGAGAAGGGGGCAACTCCATCTCAGAGCGGAGA<br>ATTTGGACAAACTTGGTCATTAGA

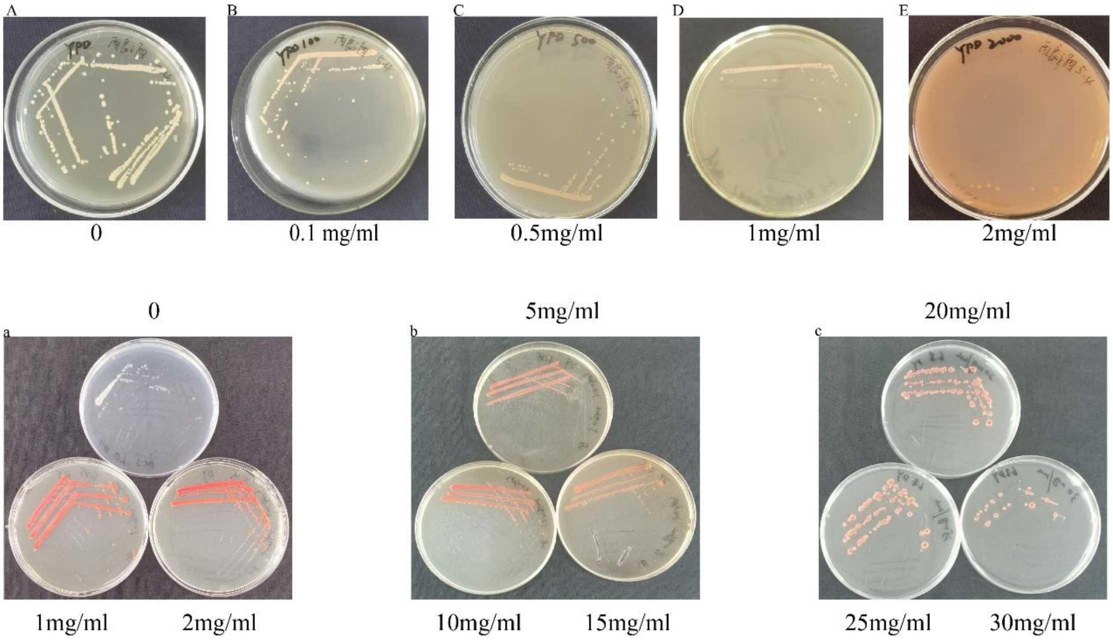

Fig. S1. Growth of *Bacillus licheniformis* and *Saccharomyces cerevisiae* in Sodium Selenite Environments of Different Concentrations.

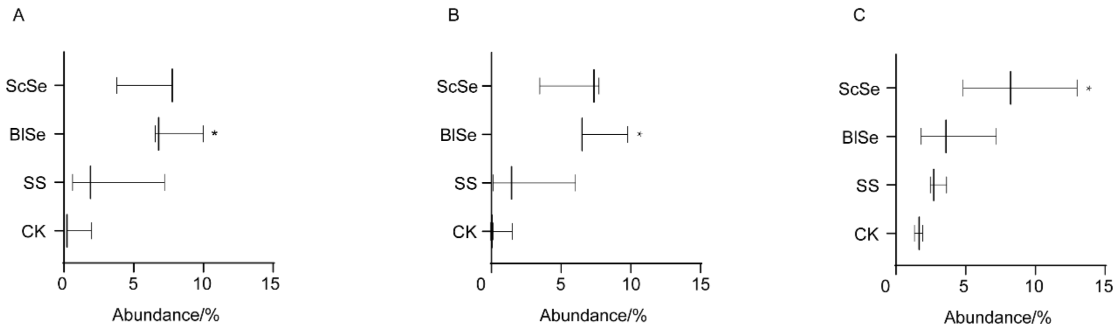

Fig. S2. Detailed abundance and differential analysis of Verrucomicrobia, *Terrimicrobium*, and *Reyranella*. A is the abundance of Verrucomicrobia in each group, B is the abundance of *Terrimicrobium* in each group, and C is the abundance of *Reyranella* in each group. “*” presents $P<0.05$ with a statistical difference.

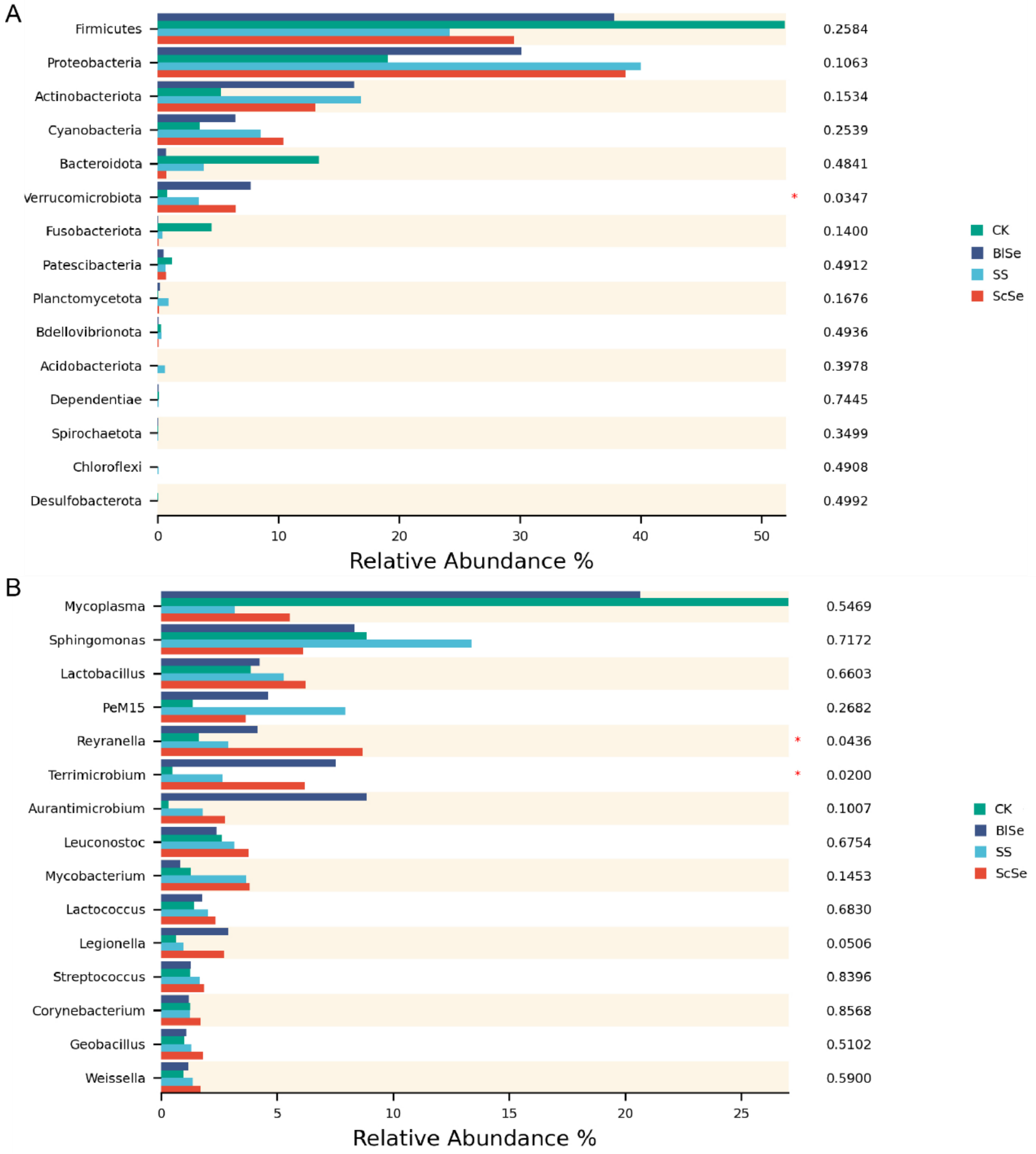

Fig. S3. Differences in abundance of individual groups of intestinal flora at the phylum level and at the genus level. A is the analysis of variance at the phylum level for each group, and B is the analysis of variance at the genus level for each group. "*" presents $P<0.05$ with a statistical difference.

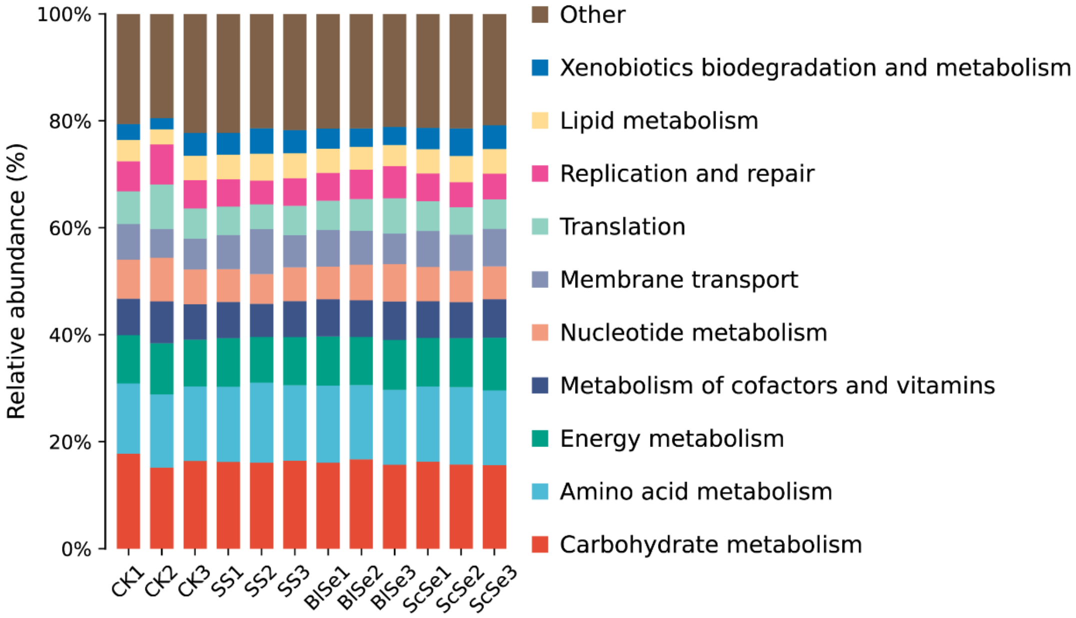

Fig. S4. The comparison of KEGG secondary function among groups.

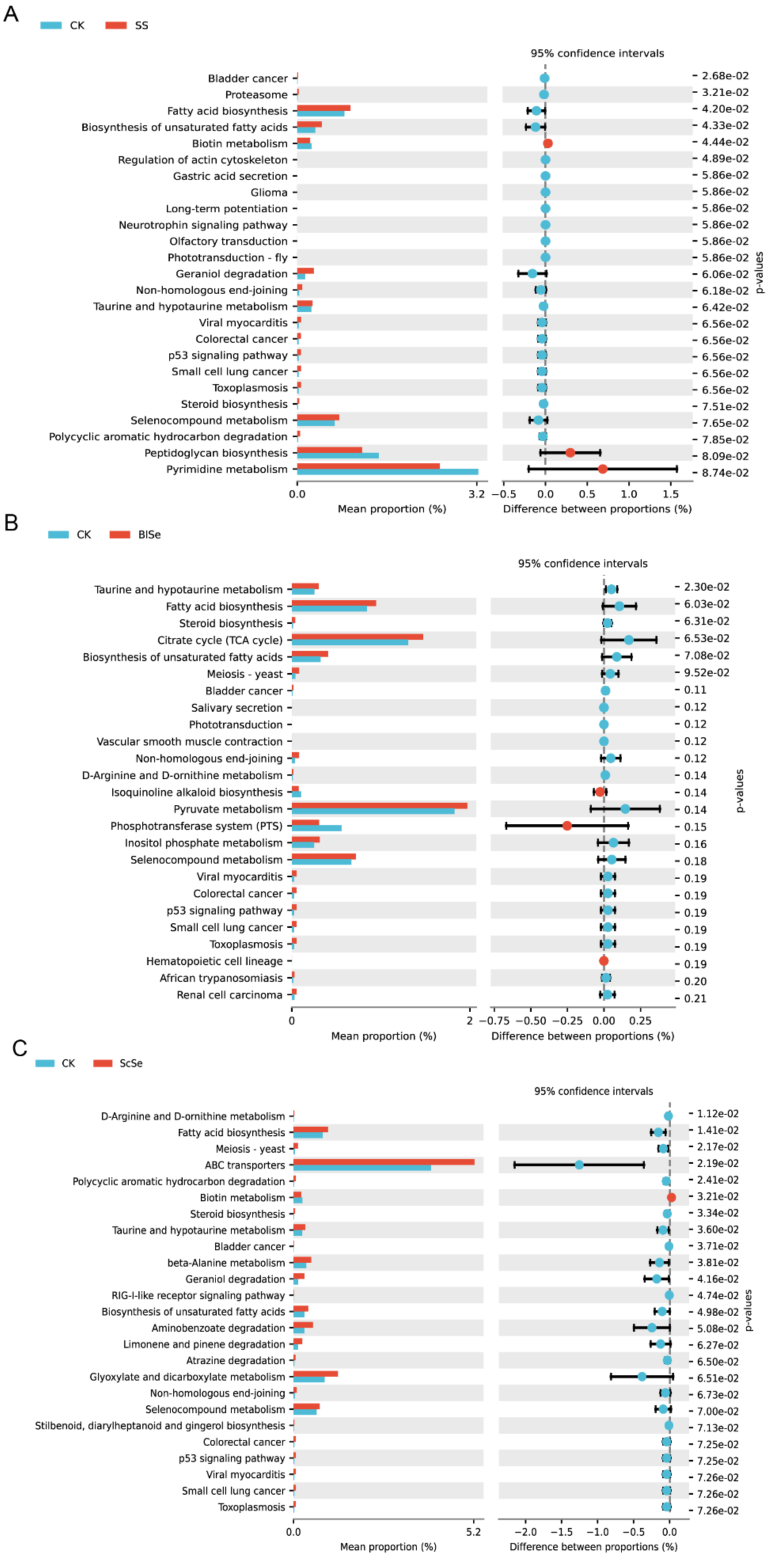

Fig. S5. Differential analysis of the abundance of each tertiary metabolic pathway between groups. A is the differential comparison between CK and SS, B is the differential comparison between CK and BlSe, and C is the differential comparison between CK and ScSe
